# The impact of gradient variable temperature fermentation on the quality of cigar tobacco leaves

**DOI:** 10.3389/fmicb.2024.1433656

**Published:** 2024-12-13

**Authors:** Qianying Zhang, Yang Huang, Hongyue An, Shuanghong Yang, Jinshan Lei, Yue Wang, Pinhe Li, Hongfei Zhang, Wen Cai, Yun Jia, Yongqiang Pang, Dongliang Li

**Affiliations:** ^1^Cigar Fermentation Technology Key Laboratory of China Tobacco, Cigar Technology Innovation Center of China Tobacco, China Tobacco Sichuan Industrial Co., Ltd., Chengdu, China; ^2^Industry Efficient Utilization to Domestic Cigar Tobacco Key Laboratory of Sichuan Province, Great Wall Cigar Factory, Shifang, China; ^3^China National Tobacco Quality Supervision and Test Centre, Zhengzhou, China; ^4^Bioengineering College, Chongqing University, Chongqing, China

**Keywords:** cigar tobacco leaf (CTL), gradient variable temperature fermentation, bacterial community, chemical component, correlation analysis

## Abstract

**Introduction:**

In order to enhance the quality of cigar tobacco leaves (CTLs), a gradient variable temperature fermentation approach was employed.

**Methods:**

The temperature gradient demonstrated a gradual increase from low temperature (35 ± 2°C) to moderate temperature (45 ± 2°C), and then to high temperature (55 ± 2°C). Each temperature gradient underwent a 10-day fermentation process, resulting in a total duration of 30 days. Changes in sensory evaluation, chemical composition, and bacterial absolute quantitative structure and function were examined throughout the process of gradient variable temperature fermentation.

**Results:**

Compared to constant temperature fermentation, gradient variable temperature fermentation improved the sensory quality of CTLs, reduced total sugar and cembrane degradation products, and increased the amino acid contents. It resulted in significant changes in bacterial quantity and function of CTLs, but had no significant effects on the richness and diversity of bacterial communities. The results of correlation analyses showed that sensory quality had significant correlation with chemical composition, which effected by predominant microbes. The gradient variable temperature fermentation process underwent a three-phase model to characterize the alterations of CTLs. Phase I (35°C) was the microbial stage, during which there was a significant decrease in both the total number and function of microorganisms. The dominant genera shifted from *Acinetobacter* to *Staphylococcus* and *Corynebacterium*, and with high reducing sugar, polyphenol compound and low sensory score. Phase II (45°C) marked a chemical stage, with an enhancement in sensory evaluation. A total of 17 chemicals significantly decreased and six increased, and the decline of microbial populations persisted. The enhanced relative abundances of four microecological hubs, namely *Staphylococcus*, *Corynebacterium*, *Oceanobacillus*, and *Bacillus*, had the potential to produce protease and lipase to the production of peptides, amino acids, and organic acid, catabolizing sugars and polyphenol compounds, through carbohydrate metabolism and amino acid metabolism, resulted an increase in sensory quality of CTLs. Phase III (55°C) indicated a relative mature stage with the highest score of sensory evaluation. Eight compositions from plamochromic pigments and polyphenol compounds exhibited gradual decreases, while relative contents of carbohydrate metabolism and amino acid metabolism increased.

**Conclusion:**

The gradient variable temperature fermentation had demonstrated a significant positive influence on the quality of CTL by providing optimal fermentation temperature for microbial growth, metabolism, and the generation of quality-related chemical compositions.

## 1 Introduction

Fermentation plays a crucial role in enhancing the quality of cigar tobacco leaves (CTLs). CTLs fermentation encompassed two distinct stages: agricultural fermentation and natural aging. Agricultural fermentation is conducted through the process of stack fermentation. For each stack, the minimum quantity of agricultural fermentation of cigar leaf is one ton. During the agricultural fermentation stage, the elevation of stack temperature typically depends on the inherent chemical, enzyme, and microbial reactions within the CTL itself. The temperature of CTLs gradually increases. Once the central stack temperature of the CTLs reaches the designated level, the stack is turned over to ensure that each leaf undergoes a uniform fermentation process from low to high temperatures, thereby improving the overall consistency of the CTLs. Following agricultural fermentation, there is a reduction in protein and starch content within the CTL leading to a significant decrease in irritation and impurities. Subsequently, these fermented CTLs undergo natural aging within cigar factory warehouses to enhance their chemical composition resulting in improved smoking characteristics and heightened aroma. The temperature in the warehouse typically ranges from 25°C to 30°C. It is a common practice for naturally aging CTLs to undergo maturation for more than 2 years in order to achieve optimal quality for utilization (Zheng et al., 2022b). However, some low-quality CTLs even after a five-year aging process, still exhibit persistent bitterness and heavy impurities, showing no improvement, which significantly hinders their suitability for use in cigar recipes.

In order to alleviate the pressure of warehouse and enhance the availability of low-quality CTLs, it is imperative to implement industrial fermentation treatment. During industrial fermentation, CTLs are typically divided into small sacks weighing 20–50 kg and placed in a controlled environment with constant temperature and humidity to facilitate the fermentation process. Industrial fermentation treatment mainly involves utilization of fermentation medium (Zhang et al., 2023b; Cai et al., 2024), and monitoring temperature and humidity levels (Di Giacomo et al., 2007; Zhang et al., 2020b). The temperature ranges utilized in industrial fermentation encompass low (35 ± 2°C), moderate (45 ± 2°C) and high (55 ± 2°C) temperature. From the initiation of the CTLs entering the fermentation room, a constant temperature is maintained throughout the duration of fermentation. The fermentation process of tea is akin to that of CTLs, serving the dual purpose of enhancing internal quality and maintaining a consistent external appearance with minimal visible alterations beyond color changes. Tea was treated using gradient variable temperature fermentation, resulting in significant improvements to its quality (Huang et al., 2022; Xia et al., 2023). Lower and moderate temperatures were conducive to the growth and metabolism of microorganisms, while high temperatures facilitated the dissemination of irritating substances (Jia et al., 2023). Hence, we proposed the utilization of gradient variable temperature fermentation technology to treat CTLs in our study. This approach aimed to enhance the overall effectiveness of CTLs treatment.

There are many microorganisms on the surface of CTLs, and the microbial communities and functions differ at different production stages (Zhang et al., 2023a). At present, there are a large number of reports on the structure of microbial communities in CTLs under different fermentation processes (Di Giacomo et al., 2007; Zhang et al., 2023a; Zheng et al., 2022a), and these previous studies have provided information on microbial communities based on microbial relative abundance. As fermentation progresses, the total number of microorganisms in CTLs gradually decreases (Du et al., 2016; Li et al., 2019). However, the increase in the relative abundance of a certain group in the microbial community of CTLs may be caused by a decrease in the absolute abundance of other microbial groups and may not really reflect the change in the absolute abundance of corresponding microbial groups (Tkacz et al., 2018; Tourlousse et al., 2017). Therefore, this study used a high-throughput absolute quantitative method in order to reveal the impact of the bacterial structural succession and functional changes during the process of the gradient variable temperature fermentation.

In this study, we assessed the impact of the gradient variable temperature fermentation on the qualities of CTLs. Furthermore, we investigated the variations in pH, chemical composition, and bacterial structure and function in CTLs during the gradient variable temperature fermentation and their interrelationships. This study offered a novel approach to enhancing the utilization efficiency of CTLs through process optimization, while also presenting a new perspective on uncovering the succession of bacterial communities.

## 2 Materials and methods

### 2.1 CTL sample

The CTLs investigated in this study were Besuki No cigar leaf, sourced from Indonesia and cultivated in 2019, which are currently stored in the cigar leaf mellowing warehouse of the Great Wall Cigar Factory.

### 2.2 CTL fermentation

The CTL in the warehouse were packaged at 50 kg per package, and a total of nine packages were selected for unpacking and mixing. The mixed CTLs were divided into 21 portions of 20 kg each, which were subsequently placed into linen bags measuring 1.1 m × 1.3 m and transported to the fermentation room for further processing. The experiment was divided into seven groups (F0, F10, F20, F30, T1, T2 and T3), with three parallel experiments conducted in each group. Group-F underwent gradient variable temperature fermentation, while group-T was subjected to constant temperature fermentation. Specifically, F0 represented unfermented raw CTLs; F10 involved fermentation at low temperature condition (35 ± 2°C, 75–85%) for 10 days; F20 included moderate temperature condition (45 ± 2°C, 75–85%) for 10 days following low temperature condition (35 ± 2°C, 75–85%) for 10 days; and F30 entailed high temperature condition (55 ± 2°C, 75–85%) for 10 days after low temperature (35 ± 2°C, 75–85%) for 10 days and moderate temperature (45 ± 2°C, 75–85%) for 10 days. On the other hand, T1 involved constant temperature and humidity fermentation under low temperatures for 30 days; T2 underwent constant temperature and humidity fermentation under moderate temperatures for 30 days; while T3 experienced constant temperature and humidity fermentation under high temperatures for 30 days. Following completion of the respective fermentations, samples were collected from each experimental group using a three-layered five-point method, followed by thorough mixing and storage at −20°C for subsequent analysis.

### 2.3 Sensory evaluation

After completion of fermentation, the tobacco leaves were ventilated, dried, and rolled into single-feed cigar samples. After the moisture was balanced, the quality characteristics of the CTLs were identified and scored by nine cigar sensory evaluation experts, and the four quality characteristics of the CTLs were scored according to 0–9 points. The evaluation indicators were aroma characteristics such as aroma volume, richness, and maturity; smoke characteristics such as irritation, softness, and fineness; aftertaste characteristics such as sweetness, cleanliness, and taste; and combustion characteristics such as flammability, grey, and condensing intensity.

### 2.4 pH and chemical compositions analysis

The pH of the samples was determined according to the tobacco industry standard and the self-established method of the National Tobacco Quality Supervision and Inspection Center (YC/T 222, 2007). Chemical composition included conventional chemical composition, plamochromic pigments, polyphenol compounds, amino acids, alkaloids, organic acids, volatile aroma components of CTLs had been detected. Continuous flow analysis was used in order to determine the total alkaloid (YC/T 468, 2013), reducing sugar, total sugar (YC/T 159, 2019), chlorine (YC/T 162, 2011) and potassium (YC/T 217, 2007) in the samples. Plamochromic pigments (YC/T 382, 2010), polyphenol compounds (YC/T 202, 2006) and amino acids in cigar tobacco were determined using high-performance liquid chromatography. Alkaloids (YC/T 383, 2010), organic acids, and volatile aroma components were determined using gas chromatography–mass spectrometry (Zhu et al., 2023).

### 2.5 Bacterial community analysis

There was a total of 21 CTL samples, and each sample was cut into pieces using sterile scissors and mixed thoroughly. Subsequently, 10.0 g of each cut CTL was placed in sterile normal saline, oscillated at 4°C for 2 h, centrifuged at 4°C and 12,000 × g for 30 min, and after this the microorganisms (sediments) were collected. The absolute quantification sequencing was conducted by Majorbio (Shanghai, China). The absolute quantitative method for microbial 16S rRNA gene involves the addition of spik-in DNA sequences with varying known copy number concentrations to the sample DNA, followed by joint PCR amplification, library construction and sequencing. The sample DNA was amplified and sequenced using the V3-V4 hypervariable region of 16S rRNA genes, forward primer 338F (5’-ACT CCT ACG GGA GGC AGC AG-3′) and reverse primer 806R (5’-GGACTACHVGGGTWTCTAAT-3′) (Xu et al., 2016). The spik-in DNA, an artificially designed and synthesized sequence with variable regions that do not align with public database nucleotide sequences, serves as the internal standard for quantification. Twelve distinct spike-in DNA samples, each with a known copy number, are diluted to specific concentrations and added to the sample DNA as templates for PCR amplification. Standard curves are then generated based on amplicon sequencing results of spik-in DNA and its theoretical absolute copy numbers. The absolute copy number of the 16S rRNA gene was calculated for each operational taxonomic unit (OTU) in the samples (Tkacz et al., 2018; Tourlousse et al., 2017).

### 2.6 Statistical analysis

The experiments were conducted in triplicate and the data was reported as the mean value accompanied by its corresponding relative standard deviation. Duncan’s SPSS software was used to analyse the differences in the data through one-way ANOVA (*p* < 0.05). BTtools was used to perform cluster analysis of the chemical components of the samples, and SIMCA software was used to perform partial least squares analysis (PLS) to analyse the correlation between the chemical components and sensory quality. Mothur was used for *α* diversity analysis of microbial data, and RDP classifier was used for species classification. Silva 132 was used as the bacterial gene reference database, and PICRUSt2 was used for bacterial function prediction. Spearman’s correlation coefficient was used to establish a microbial symbiosis model, and the correlation between bacteria and chemical composition was analysed. Gephi software was used for the network visualization.

## 3 Results

### 3.1 Profiles of sensory evaluation

**Figure 1:**
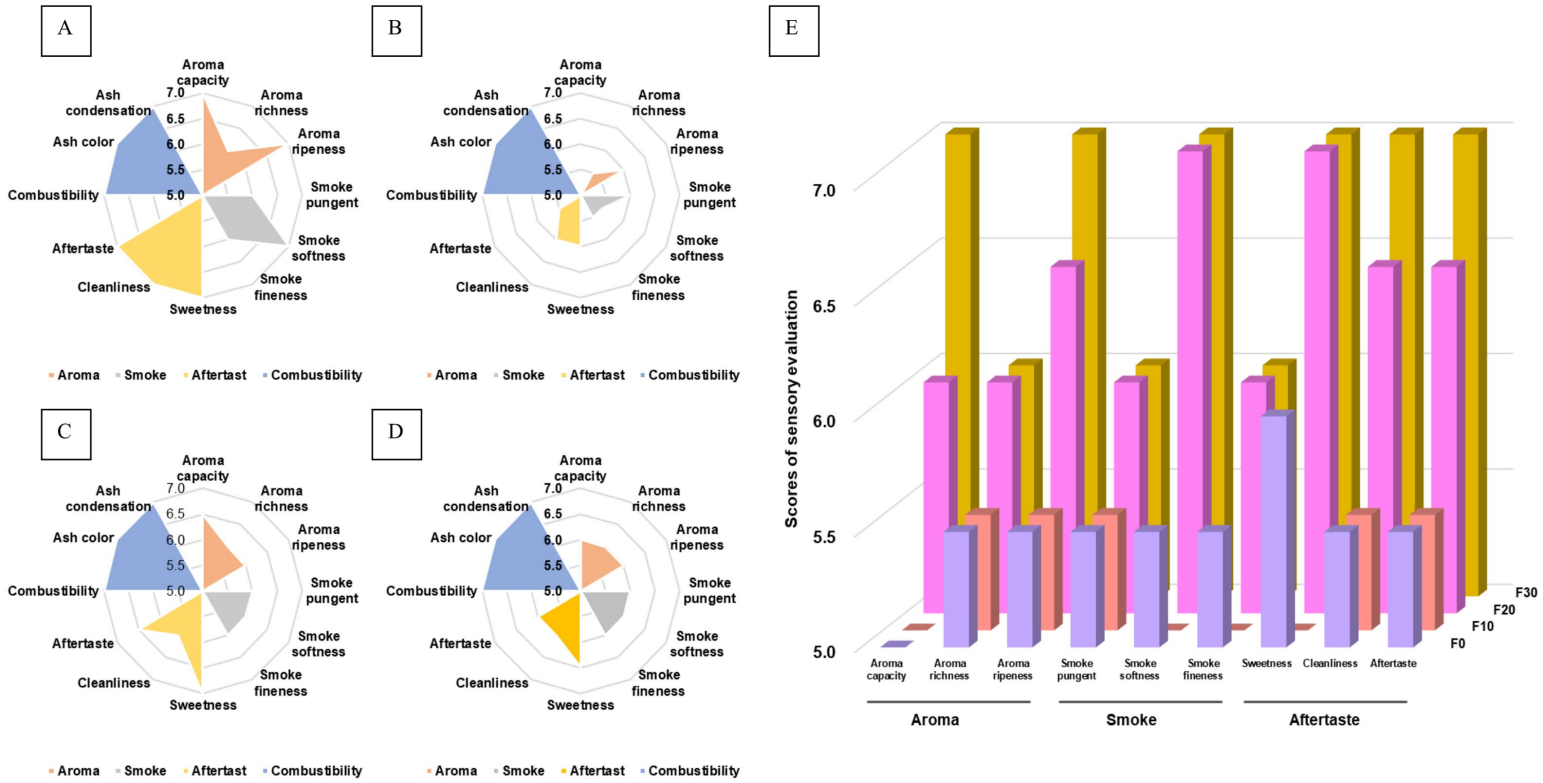
Plot of the sensory evaluation results of the CTLs. The sensory characteristics of samples were evaluated after undergoing fermentation at gradient varable temperature **(A)**, and consistent low **(B)**, moderate **(C)**, and high **(D)** temperatures for a duration of 30 days. The sensory characteristics of during fermentation at various temperatures **(E)**. Samples labeled F0, F10, F20, and F30 underwent fermentation at various temperatures over the course of 0, 10, 20, and 30 days.

The results of the sensory evaluation of the fermented CTLs are shown in Figure 1. As shown in Figure 1, the different fermentation methods had no significant effects on the combustion characteristics of tobacco leaves (*p* > 0.05). The sensory evaluation results of F30 were better than those of the constant temperature fermentation group (*p* < 0.05), which showed that the aroma quantity and maturity in aroma characteristics, softness in the smoke characteristics, and cleanliness and taste sense in the aftertaste characteristics were significantly improved. The sensory quality of moderate temperature fermented CTLs (T2) was found to be significantly higher than that of high temperature fermented CTLs (T3). The sensory quality of low temperature fermented CTLs (T1) was deemed the least preferred.

Further analysis of the sensory quality changes in CTLs during the gradient variable temperature fermentation, it was observed that the sensory quality initially decreased and then subsequently improved as the fermentation progressed. At the end of fermentation, the sensory quality of the CTLs significantly improved (*p* < 0.05). Following 10 days of fermentation (F10), the softness, fineness, and sweetness of the CTLs were found to be lower compared to unfermented CTLs (F0). However, after 20 days of fermentation (F20), there was a notable improvement in sensory indices. Subsequent to 30 days of fermentation (F30), there was further enhancement in aroma, maturity, cleanliness, and taste of the CTLs.

### 3.2 Profiles of pH and chemical composition

The pH and conventional chemical composition of the fermented CTLs are shown in Table 1. In terms of the conventional chemical composition, the content of total alkaloids in CTLs significantly decreased after fermentation (*p* < 0.05), and T2 had the highest total alkaloid content among the different fermentation methods (F30, T1, T2, and T3). This indicated that fermentation could significantly reduce the total alkaloid content in CTLs, and fermentation temperature had a significant impact on it. The total sugar content in CTLs (T1, T2, and T3) was found to significantly decrease with an increase in the fermentation temperature (*p* < 0.05), and the total sugar content in these three groups of tobacco leaves was lower than that in unfermented tobacco leaves (F0). The potassium ion content in F30 was significantly higher than that in the thermostatically fermented tobacco leaves. The pH, total alkaloid, total sugar, and chloride ion contents of unfermented tobacco leaves (F0) were the highest among all samples. During gradient variable temperature fermentation, the reducing sugar content in tobacco leaves first increased and then decreased. No reducing sugar or total sugar was detected in the tobacco leaves (F20 and F30) after 20 days of fermentation. The potassium ion content increased gradually during fermentation, but there was no significant difference between F20 and F30.

**Table 1:** The pH and routine chemical compositions of CTLs.

Sample	pH	Routine chemical compositions (%)				
		Total alkaloid	Reducing sugar	Total sugar	Cl	K
F0	6.02 ± 0.01a	2.69 ± 0.00a	2.26 ± 0.04b	3.30 ± 0.07a	1.06 ± 0.00a	4.01 ± 0.01c
F10	5.93 ± 0.01d	2.55 ± 0.00c	2.78 ± 0.10a	3.18 ± 0.09b	1.01 ± 0.00d	4.19 ± 0.14b
F20	5.93 ± 0.01d	2.39 ± 0.00f	–	–	1.02 ± 0.00c	4.34 ± 0.01a
F30	5.95 ± 0.01c	2.46 ± 0.00d	–	–	1.05 ± 0.00b	4.42 ± 0.02a
T1	5.92 ± 0.01d	2.42 ± 0.01e	–	2.40 ± 0.02c	1.01 ± 0.00d	4.06 ± 0.01c
T2	5.97 ± 0.01b	2.58 ± 0.00b	–	1.78 ± 0.01d	0.99 ± 0.00e	3.97 ± 0.01c
T3	5.93 ± 0.01d	2.29 ± 0.01 g	–	1.41 ± 0.03e	1.01 ± 0.00d	4.05 ± 0.00c

“–” meant not detected. Different letters indicate significant differences between different groups (*P* < 0.05). F0, F10, F20, and F30 were subjected to fermentation at the gradient various temperatures over the course of 0, 10, 20, and 30 days. T1, T2, and T3 underwent fermentation at consistent low, moderate, and high temperatures for a duration of 30 days.

**Figure 2:**
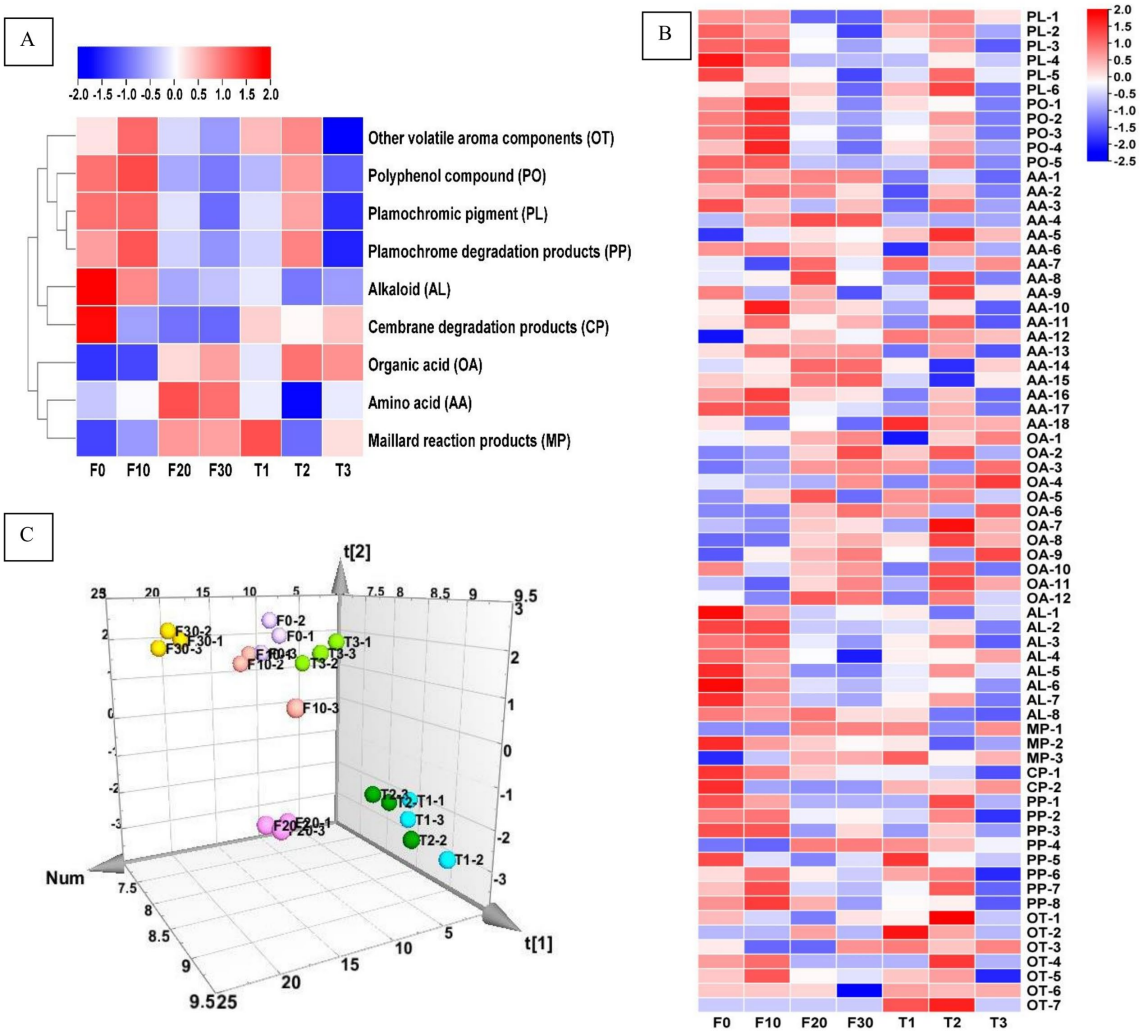
The heat map of the chemical components (**A**), chemical substances (**B**), and PLS three-dimensional score heat map of the samples based on chemical substances (**C**). F0-1, F0-2, and F0-3; F10-1, F10-2, and F10-3; F20-1, F20-2, and F20-3; F30-1, F30-2, and F30-3; T1-1, T1-2, and T1-3; T2-1, T2-2, and T2-3; T3-1, T3-2, and T3-3 represented three simultaneous samples of F0, F10, F20, F30, T1, T2, and T3. F0, F10, F20, and F30 were subjected to fermentation at gradient variable temperatures over the course of 0, 10, 20, and 30 days. T1, T2, and T3 underwent fermentation at consistent low, moderate, and high temperatures for a duration of 30 days. PL-1 indecated Neoxanthin; PL-2, Violaxanthin; PL-3, Lutein; PL-4, Chlorophyll B; PL-5, Chlorophyll A; PL-6, *β*-Carotene; PO-1, Neochlorogenic acid; PO-2, Chlorogenic acid; PO-3, Cryptochlorogenic acid; PO-4, Scopoletin; PO-5, Rutin; AA-1, Aspartic acid; AA-2, Serine; AA-3, Glutamic acid; AA-4, Glycine; AA-5, Histidine; AA-6, Arginine; AA-7, Threonine; AA-8, Alanine; AA-9, Proline; AA-10, 4-Aminobutyric acid; AA-11, Cystine; AA-12, Tyrosine; AA-13, Valine; AA-14, Methionine; AA-15, Lysine; AA-16, Isoleucine; AA-17, Leucine; AA-18, Phenylalanine; OA-1, Oxalic acid; OA-2, Tartaric acid; OA-3, Formic acid; OA-4, Malic acid; OA-5, Malonic acid; OA-6, Ketovaleric acid; OA-7, Lactic acid; OA-8, Acetic acid; OA-9, Citric acid; OA-10, Maleic acid; OA-11, Fumaric acid; OA-12, Succinic acid; AL-1, Nicotine; AL-2, Nornicotine; AL-3, Myosmine; AL-4, Pseudoequine; AL-5, β-Diene nicotine; AL-6, Anatabine; AL-7, 2, 3-Bipyridine; AL-8, Cotinine; MP-1, 4-Cyclopentene-1,3-dione; MP-2, 3-Methylpentanoic acid; MP-3, 3-Ethyl-3,4-dihydro-2(1H)-quinoxalinone; CP-1, Solanone; CP-2, Thunbergol; PP-1, Dihydroactindiolide; PP-2, Megastigmatrienone; PP-3, 4-(3-Hydroxy-1-butenyl)-3,5,5-trimethyl-2-cyclohexen-1-one; PP-4, 6-(3-Hydroxy-1-butenyl)-1,5,5-trimethyl-7-oxabicyclo[4.1.0]heptan-3-ol; PP-5, 6-Hydroxy-4,4,7a-trimethyl-5,6,7,7a-tetrahydrobenzofuran-2(4H)-one; PP-6, 6,10,14-Trimethyl-2-pentadecanone; PP-7, Phytyl acetate; PP-8, Phytol; OT-1, 2,4-Dimethyl-1-heptene; OT-2, 2-Methylbutanoic acid; OT-3, 3-Methoxy-3-methyl-1-butanol; OT-4, Hexadecanoic acid methyl ester; OT-5, 1,5,9-Trimethyl-12-(1-methylethyl)-4,8,13-cyclotetradecatriene-1,3-diol; OT-6, Isoaromadendrene epoxide; OT-7, Sclareolide.

The contents of plamochromic pigments (PL), polyphenol compounds (PO), amino acids (AA), organic acids (OA), alkaloids (AL), maillard reaction products (MP), cembrane degradation products (CP), plamochromic degradation products (PP), and other volatile aroma compounds (OT) were shown in Figure 2A, Table 2, and Supplementary Table 1. It could be concluded that fermentation (with varying or constant temperature) could achieve the accumulation of OA, and MP, as well as a reduction in the content of PL, AL, and CP. In terms of constant temperature fermentation, as the temperature increased, the contents of PL, PO, and PP in CTLs first increased and then decreased, and the total amount of various substances was the highest in T2. In the process of variable temperature fermentation, the total content of PO and PP first increased and then decreased with the progress of fermentation, and the contents were the highest on the 10th day of fermentation (F10). The total amounts of AA, OA, and MP increased gradually during fermentation, whereas the total amounts of PL, AL, CP, and PO showed the opposite trend.

**Table 2:** The chemical composition content of CTLs.^†^

Compounds	No.	Samples						
		F0	F10	F20	F30	T1	T2	T3
Plamochromic pigment	PL	203.77 ± 15.82a	206.54 ± 3.06a	155.59 ± 1.38b	128.93 ± 2.16c	155.72 ± 2.83b	187.65 ± 17.37a	117.16 ± 24.39c
Polyphenol compound	PO	2633.49 ± 6.29b	2982.07 ± 27.84a	1285.17 ± 15.77d	1108.70 ± 49.37e	1351.70 ± 43.05d	2307.48 ± 69.50c	1012.95 ± 61.27f
Amino acid	AA	64543.05 ± 3778.57	69141.40 ± 3999.71	87640.00 ± 5169.79	84031.51 ± 3606.00	67859.28 ± 3661.24	49866.81 ± 3189.34	67755.30 ± 3431.09
Organic acid	OA	116.87 ± 4.02d	119.97 ± 10.15d	163.73 ± 14.00bc	177.01 ± 2.42ab	150.07 ± 16.04c	188.79 ± 5.04a	180.69 ± 4.83ab
Alkaloid	AL	21888.00 ± 2893.89a	19793.76 ± 394.17ab	17070.81 ± 288.78b	17403.59 ± 33.91b	17885.87 ± 491.80b	16481.19 ± 3362.65b	16935.59 ± 455.50b
Maillard reaction products	MP	209.74 ± 14.23c	224.99 ± 22.60bc	265.68 ± 7.48a	263.77 ± 16.60a	281.98 ± 28.79a	216.75 ± 12.51c	250.97 ± 9.08ab
Cembrane degradation products	CP	162.23 ± 58.25	93.81 ± 29.83	87.22 ± 62.13	85.73 ± 60.30	117.69 ± 67.76	110.20 ± 69.70	119.70 ± 51.58
Plamochrome degradation products	PP	1250.61 ± 70.32ab	1322.54 ± 111.68a	1121.79 ± 11.33bc	1070.94 ± 42.88 cd	1123.52 ± 114.87bc	1277.28 ± 53.24a	979.86 ± 39.27d
Other volatile aroma components	OT	2073.39 ± 246.05abc	2309.10 ± 235.85a	1956.49 ± 74.97bc	1853.84 ± 120.14 cd	2149.33 ± 194.69abc	2247.79 ± 71.38ab	1615.24 ± 80.12d

^†^The chemical compounds contents were measured in μg/g. Different letters indicated significant differences between different groups (*P* < 0.05). F0, F10, F20, and F30 were subjected to fermentation at gradient variable temperatures over the course of 0, 10, 20, and 30 days. T1, T2, and T3 underwent fermentation at consistent low, moderate, and high temperatures for a duration of 30 days.

Further analysis was conducted on the effect of fermentation temperature (Figure 2B). There were significant differences between different stages of gradient variable temperature fermentation and constant temperature fermentation. In the gradient variable temperature fermentation, F10 exhibited chemical components richness, with the contents of 5 decreased and 10 increased, especially in PO; there were 17 decreased and 6 increased in F20, and 8 decreased in F30. F20 and F30 both had increased types and contents of AA and OA. This indicated that substances were constantly changing during the fermentation process. In constant temperature fermentation, T2 had the most abundant substance, with the majority being AA, PL and PO, and T3 had the least chemical composition, indicating that high temperature constant temperature fermentation was not conducive to substance accumulation.

### 3.3 Correlation analysis of chemical composition and sensory quality

SIMCA-P software was used to perform PLS analysis with the chemical composition as the X variable and the total score of the sensory evaluation as the Y variable. Two principal components were identified. The interpretability R2X of the model for the X variable was 90.6%, interpretability R2Y of the Y variable was 99.7%, and the predicted Q2 value was 99.6%. By combining these indicators, the PLS model was found to be suitable for this study.

A three-dimensional PLS score diagram of the fermented tobacco leaf samples is shown in Figure 2C. Samples from groups T1 and T2 were present in the lower right of the 3D chart; samples from groups T3, F0, and F10 were present in the upper part of the 3D score chart; samples from group F20 were present in the lower middle part; and samples from group F30 were present in in the upper left part.

The contribution of each variable to the total score of sensory evaluation was examined and recorded as VIP value in PLS, where VIP value >1 indicates that the variable is an “important” variable, and <0.5 indicates that it is a “non-important” variable. The VIP values and correlation coefficients of “important” variables in the PLS fitting model of this study are shown in Table 3. A total of 48 chemical compositions were identified. Among them, three plamochromic pigments, four polyphenolic compounds, 14 amino acids, nine organic acids and eight alkaloids, as well as two Maillard reaction products, one cembrane degradation product and five plamochrome degradation products were important contributing variables with a significant positive effect on the total score of sensory evaluation of CTLs. These substances accounted for 70% of the total substances measured, indicating that the chemicals in tobacco composition were closely related to flavor. In other words, changes in chemical composition could alter the final smoking quality of CTLs.

**Table 3:** VIP values and coefficient values between chemical compounds and total scores of sensory evaluation.^†^

Variables		VIP values [1]	Coefficient values [1]	VIP values [2]	Coefficient values [2]
Violaxanthin	PL-2	1.0254	0.0141	1.0226	0.0095
Lutein	PL-3	1.0531	0.0145	1.0487	0.011
β-Carotene	PL-6	1.0697	0.0147	1.0635	0.0143
Neochlorogenic acid	PO-1	1.0625	0.0146	1.0569	0.0126
Chlorogenic acid	PO-2	1.0242	0.0141	1.0241	0.0078
Cryptochlorogenic acid	PO-3	1.0686	0.0147	1.0627	0.0131
Scopoletin	PO-4	1.0699	0.0147	1.0639	0.0134
Aspartic acid	AA-1	1.0587	0.0146	1.0526	0.0154
Serine	AA-2	1.0677	0.0147	1.0614	0.0145
Glutamic acid	AA-3	1.0186	0.014	1.0132	0.0122
Histidine	AA-5	1.0656	0.0147	1.0613	0.0187
Arginine	AA-6	1.0593	0.0146	1.0532	0.0138
Threonine	AA-7	1.0389	0.0143	1.0373	0.0201
Alanine	AA-8	1.0717	0.0147	1.0658	0.0165
Proline	AA-9	1.0615	0.0146	1.0554	0.0157
4-Aminobutyric acid	AA-10	1.0556	0.0145	1.0497	0.0131
Tyrosine	AA-12	1.0533	0.0145	1.0494	0.0187
Valine	AA-13	1.0526	0.0145	1.0465	0.0142
Lysine	AA-15	1.0731	0.0148	1.0676	0.0174
Isoleucine	AA-16	1.0462	0.0144	1.0415	0.0113
Leucine	AA-17	1.0243	0.0141	1.0235	0.0081
Oxalic acid	OA-1	1.0837	0.0149	1.0788	0.0184
Tartaric acid	OA-2	1.0333	0.0142	1.0286	0.0176
Malic acid	OA-4	1.084	0.0149	1.0792	0.0186
Lactic acid	OA-7	1.0484	0.0144	1.0448	0.0189
Acetic acid	OA-8	1.0252	0.0141	1.0273	0.0219
Citric acid	OA-9	1.074	0.0148	1.0714	0.0202
Maleic acid	OA-10	1.0828	0.0149	1.0772	0.0173
Fumaric acid	OA-11	1.0833	0.0149	1.0792	0.0192
Succinic acid	OA-12	1.0783	0.0148	1.0746	0.0195
Nicotine	AL-1	1.0722	0.0147	1.066	0.0154
Nornicotine	AL-2	1.0697	0.0147	1.0637	0.0134
Mysmine	AL-3	1.0693	0.0147	1.0635	0.0131
Pseudoequine	AL-4	1.0461	0.0144	1.0403	0.0129
β-diene nicotine	AL-5	1.0202	0.014	1.0193	0.0082
Neonicotinine	AL-6	1.0801	0.0149	1.0738	0.0156
2,3-Bipyridine	AL-7	1.0664	0.0147	1.0607	0.0127
Cotinine	AL-8	1.0753	0.0148	1.0692	0.0163
3-Methylpentanoic acid	MP-2	1.0669	0.0147	1.0607	0.0148
3-Ethyl-3,4-dihydro-2(1H)-quinoxalinone	MP-3	1.0781	0.0148	1.0751	0.02
Solanone	CP-1	1.0507	0.0144	1.0456	0.0118
6-(3-Hydroxy-1-butenyl)-1,5,5-trimethyl-7-oxabicyclo[4.1.0]heptan-3-ol	PP-4	1.0844	0.0149	1.0798	0.0188
6-Hydroxy-4,4,7a-trimethyl-5,6,7,7a-tetrahydrobenzofuran-2(4H)-one	PP-5	1.0803	0.0149	1.0742	0.0163
6,10,14-Trimethyl-2-pentadecanone	PP-6	1.0754	0.0148	1.0691	0.0151
Phytyl acetate	PP-7	1.0773	0.0148	1.071	0.0146
Phytol	PP-8	1.0753	0.0148	1.0691	0.0144
2,4-Dimethyl-1-heptene	OT-1	1.0422	0.0143	1.0362	0.0149
4,8,13-Cyclotetradecatriene-1,3-diol, 1,5,9-trimethyl-12-(1-methylethyl)-	OT-5	1.0742	0.0148	1.068	0.0143

^†^Means significance at the level of 0.05. The coefficient is significant (*p* < 0.05). VIP values [1] and VIP values [2] represent the VIP of the first and second principal components, respectively, and the coefficient values [1] and [2] represent the coefficient values of the first and second principal components, respectively.

### 3.4 Overview of microbial community

**Figure 3:**
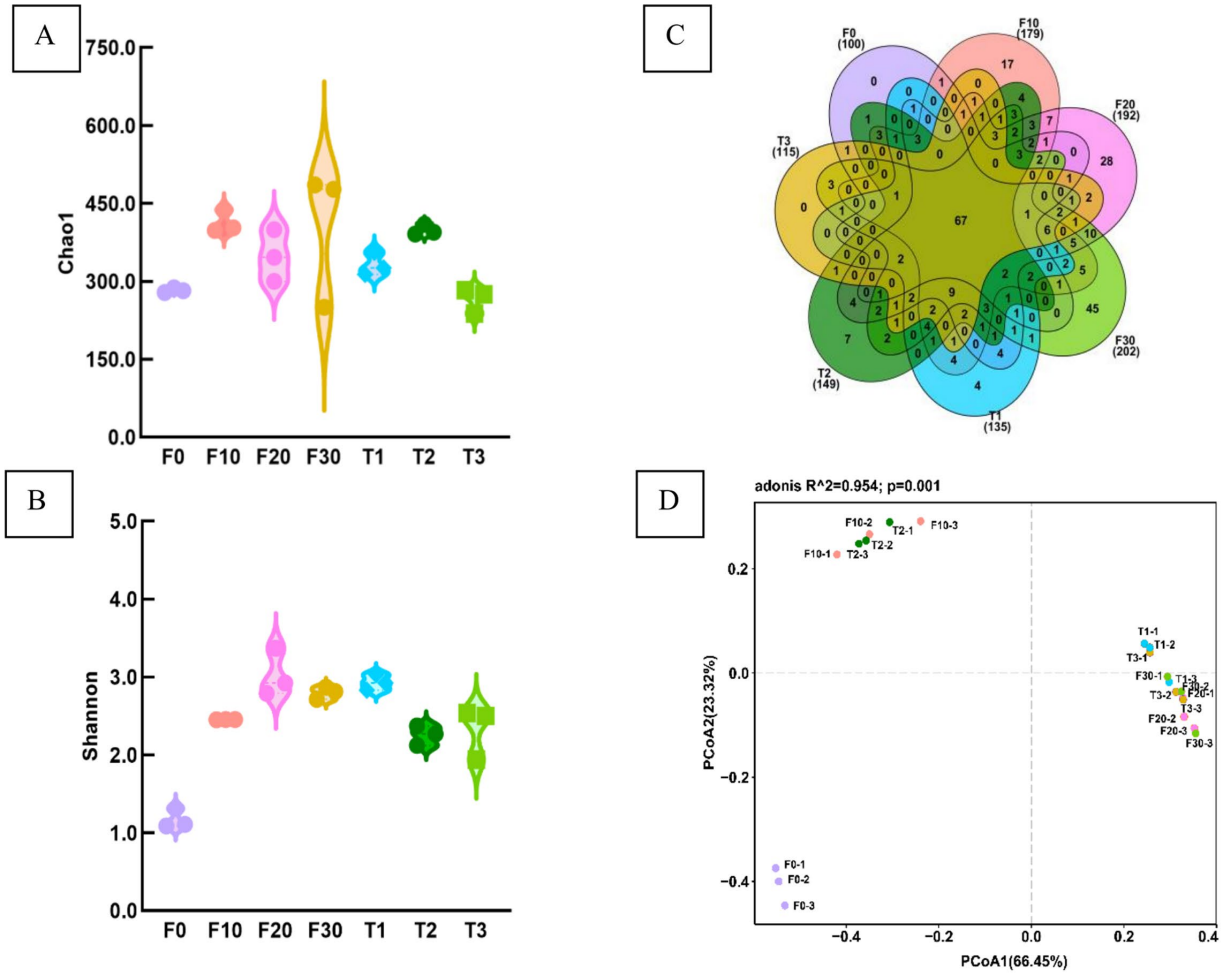
Bacterial diversity index in the CTLs. The Chao1 index **(A)**, Shannon index **(B)**, Venn diagram of OTU number **(C)**, and PCoA analysis **(D)**. F0, F10, F20, and F30 were subjected to fermentation at gradient variable temperature over the course of 0, 10, 20, and 30 days. T1, T2, and T3 underwent fermentation at consistent low, moderate, and high temperatures for a duration of 30 days.

The coverage rate of 1.0 for all samples, indicated that the sequencing results accurately reflect the true characteristics of the samples. Analysis of Chao1 and Shannon indices revealed no significant differences in bacterial richness and diversity among the CTL samples (*p* > 0.05) (Figures 3A,B).

A Venn diagram was used to analyse the number of common and unique OTUs in the CTLs (Figure 3C), showing a total number of 67 OTUs across all samples, with four, seven, and zero unique OTUs in T1, T2, and T3, respectively. Additionally, there were 17, 28, and 45 OTUs in F10, F20, and F30, respectively.

PCoA based on distance matrix was used to analyse the *β*-diversity of the samples (Figure 3D). The results indicated that significant variations in the composition of bacterial communities in the samples were attributed to different temperatures and fermentation durations. The samples were categorized into three groups: F0 was positioned in the lower left quadrant of the figure; F10 and T2 were situated in the upper left quadrant; while T1, T3, F20, and F30 occupied the central region extending toward the right side of the figure.

### 3.5 Changes in microbial flora structure and symbiotic model analysis

**Figure 4:**
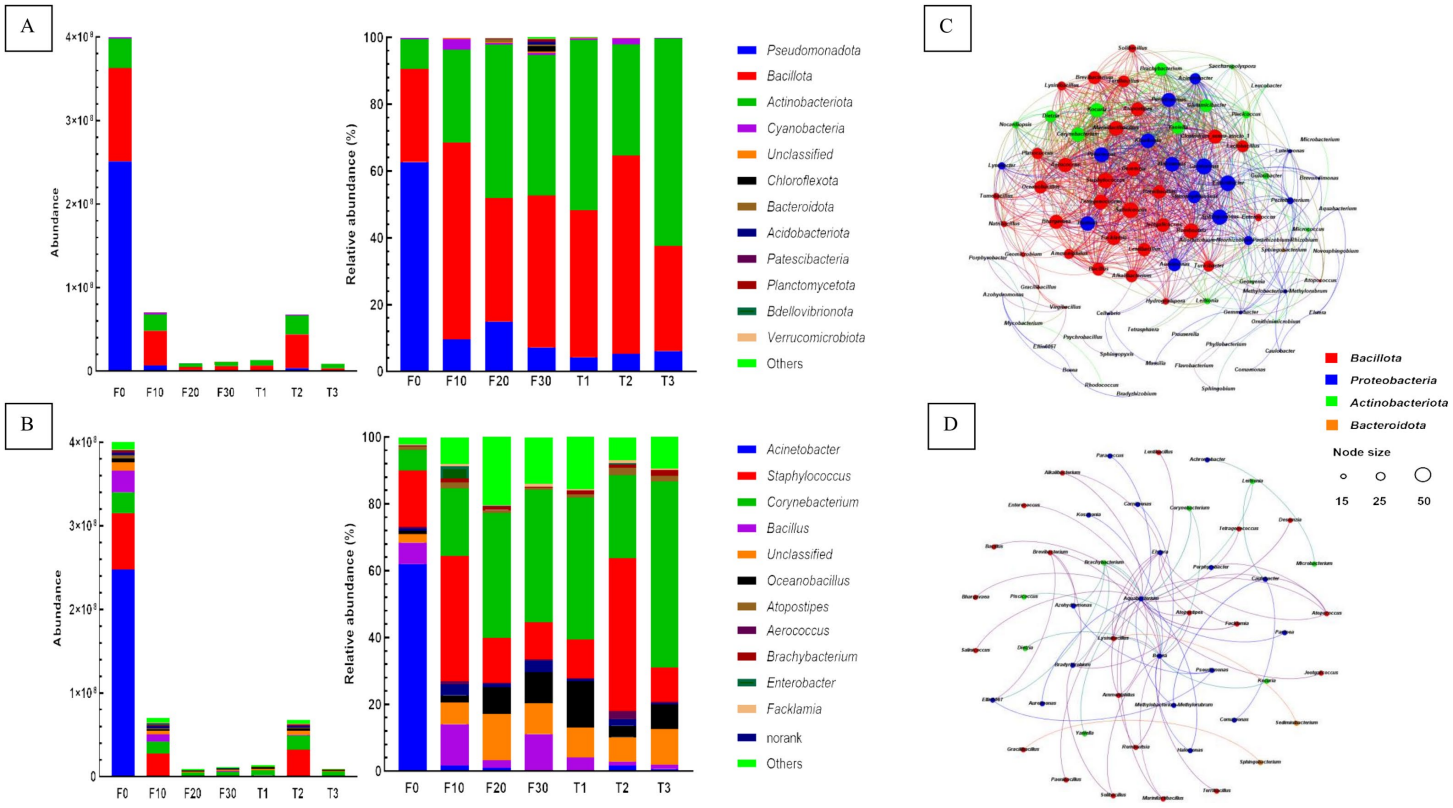
Plot of bacterial communities and network of co-occurring microbial genera. The absolute and relative abundance of bacterial communities at the phylum **(A)** and genus **(B)** levels were assessed. The correlation network of bacterial co-occurrence, encompassing both positive **(C)** and negative **(D)** associations. F0, F10, F20, and F30 were subjected to fermentation at gradient variable temperature over the course of 0, 10, 20, and 30 days. T1, T2, and T3 underwent fermentation at consistent low, moderate, and high temperatures for a duration of 30 days.

The absolute bacterial copy number was determined by referencing the absolute copy number of internal reference genes. The abundance of bacteria in CTLs at the phylum level is shown in Figure 4A. The top three phyla in all samples were *Pseudomonadota*, *Bacillota*, and *Actinobacteriota*. They were 6.5 × 10^5^–2.5 × 10^8^ copies, 2.9 × 10^6^–1.1 × 10^8^ copies and 4.2 × 10^6^–3.5 × 10^7^ copies, respectively. When compared to F0, the absolute copy number of bacterial genes in all other CTLs decreased significantly at the phylum level, among which *Proteobacteria* decreased the most, followed by *Bacillota* and *Actinobacteriota*. In the samples F10 and T2, the decrease in the three phyla was the smallest. During variable temperature fermentation, the absolute copy numbers of the three phyla decreased gradually. The relative abundances of *Pseudomonadota*, *Bacillota*, and *Actinobacteriota* in the same sample were 97.4 to 99.9%. When compared to unfermented leaves, the relative abundance of *Bacteroidota* in all other CTLs decreased significantly, and the relative abundances of *Bacillota* and *Actinobacteriota* increased significantly.

The abundance of bacteria in CTLs at the genus level is shown in Figure 4B. In terms of absolute abundance, *Acinetobacter*, *Staphylococcus*, *Corynebacterium*, *Bacillus*, and *Oceanobacillus*, five most prevalent genera, had different contents in all samples. They were 2.1 × 10^4^–2.5 × 10^8^ copies, 9.4 × 10^5^–6.7 × 10^7^ copies, 3.5 × 10^6^–2.5 × 10^7^ copies, 1.1 × 10^5^–2.6 × 10^7^ copies and 4.5 × 10^6^–6.7 × 10^5^ copies. When compared with the unfermented samples, the absolute copy number of bacterial genes in all other CTLs decreased significantly at the genus level, and the decrease was greatest in *Acinetobacter*. Further calculations of the relative abundance of each phylum in the same sample showed that *Acinetobacter*, *Staphylococcus*, *Corynebacterium*, *Bacillus*, and *Oceanobacillus* were the dominant bacterial genera in the CTLs, and their relative abundance in all samples ranged from 62.2 to 92.5%. When compared to the unfermented tobacco leaves, the relative content of *Acinetobacter* in the remaining fermented samples decreased significantly, whereas *Corynebacterium* showed the opposite trend. The relative content of *Staphylococcus* was significantly higher than that of other samples, and the relative content of *Oceanobacillus* was highest in the samples subjected to constant temperature and low-temperature fermentation.

The Spearman correlation coefficient (*p* < 0.01) was used in order to establish the correlation among the top 100 dominant microorganisms, and the co-occurrence patterns were analysed. A total of 89 microbial genera were identified and 1,068 were significantly correlated (Figures 4C,D). Microbial genera were mainly distributed in *Pseudomonadota*, *Bacillota*, *Actinobacteriota*, and *Bacteroidota*, accounting for 38.2, 37.1, 22.5, and 2.3% of the total nodes, respectively. The genera with a significant number of relationships >40 are defined as hubs or keystone species, with 31 genera, including *Atopostipes*, *Aureimonas*, *Bacillus*, *Brachybacterium*, *Jeotgalicoccus*, *Lentibacillus*, *Oceanobacillus*, *Pantoea*, *Pseudomonas*, *Salinicoccus*, *Sphingomonas*, *Stenotrophomonas*, *Tetragenococcus*, et al.

Correlations between 49 bacterial genera and 68 isolates were tested for negative correlations. Microbial genera were mainly distributed in *Pseudomonadota*, *Bacillota*, *Actinobacteriota*, and *Bacteroidota*, accounting for 42.9, 36.7, 16.3, and 4.1% of the total nodes, respectively. The top seven bacterial genera with significant relationships were *Aquabacterium*, *Bosea*, *Elstera*, *Lysinibacillus*, *Brachybacterium*, *Bradyrhizobium*, and *Caulobacter*, which showed significant negative correlations with 26, 15, 7, 6, 5, 5, and 5, respectively.

### 3.6 Changes in microbial metabolic pathways

**Figure 5:**
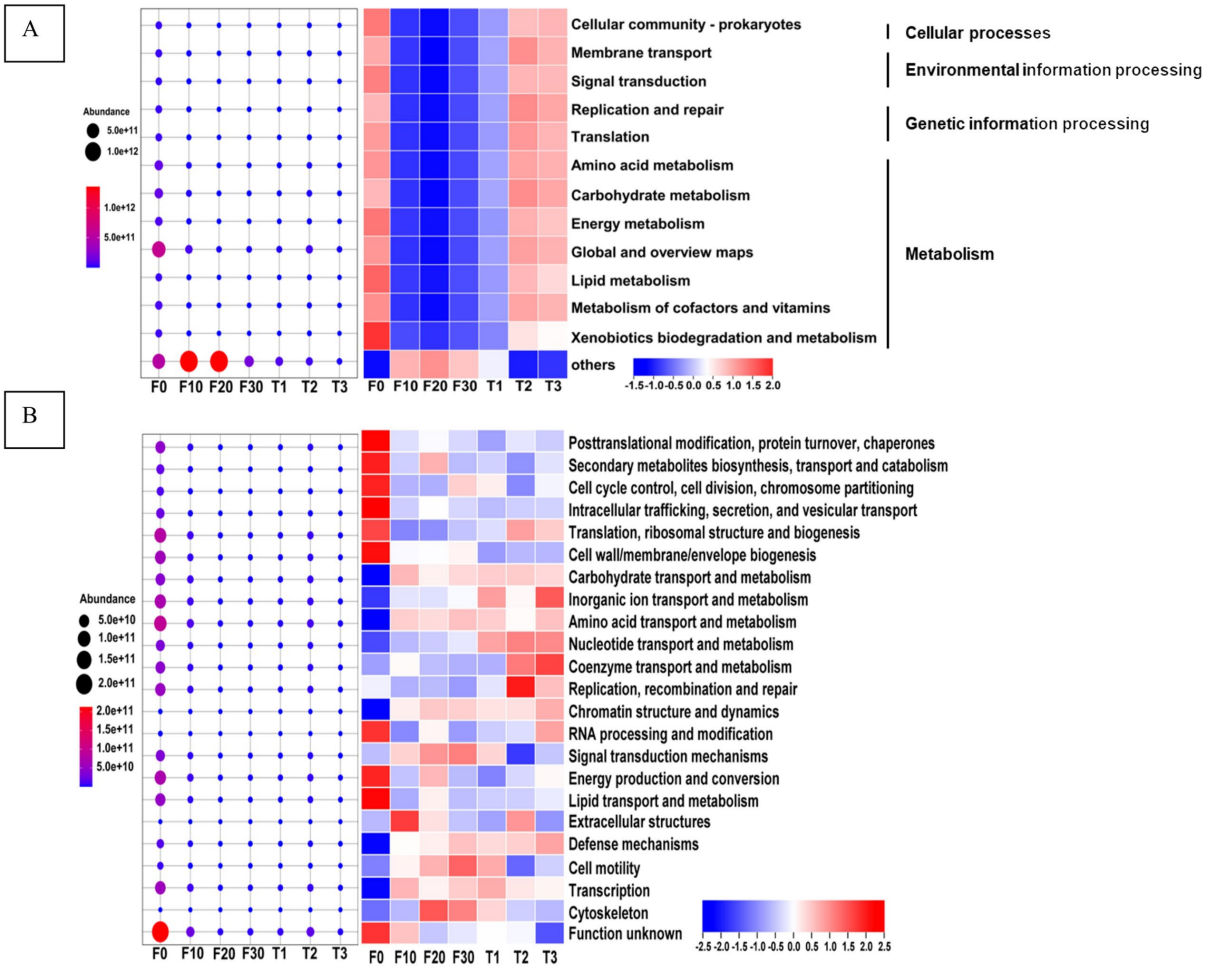
Function prediction of bacteria of the CTLs. The prediction function obtained by comparison between bacteria and KEGG database **(A)**, the prediction function obtained by comparison between bacteria and COG database **(B)**, the absolute content diagram is on the left and the relative content diagram is on the right. F0, F10, F20, and F30 were subjected to fermentation at gradient variable temperature over the course of 0, 10, 20, and 30 days. T1, T2, and T3 underwent fermentation at consistent low, moderate, and high temperatures for a duration of 30 days.

PICRUSt2 was used to compare the Cluster of Orthologous Group (COG) and Kyoto Encyclopedia of Genes and Genomes (KEGG) databases and to make functional predictions of microorganisms, as shown in Figure 5. When compared to the unfermented samples, the bacterial function of the fermented samples was significantly reduced. As gradient variable temperature fermentation progresses, bacterial function gradually decreases. When compared with constant temperature fermentation, the bacterial function was the lowest at the end of gradient variable temperature fermentation. The bacterial metabolic pathway information of the fermented CTLs was obtained by functional comparison with the KEGG database (Figure 5A). At the metabolic level, microbial functional genes in all samples were mainly concentrated in cellular processes, environmental information processing, and genetic information processing, and metabolism, of which metabolism was the strongest. At the secondary metabolic level, global and overview maps showed the highest relative and absolute abundances in all samples. The second pathway involved carbohydrate and amino acid metabolism. In terms of the relative abundance of bacterial metabolism, F0, T2, and T3 exhibited higher levels compared to the other samples.

Functional information on the bacterial proteins in fermented CTLs was obtained by functional comparison with the COG database, as shown in Figure 5B. Microbial protein functions with a relative content of more than 5% in the sample mainly included translation, ribosomal structure and biogenesis, replication, recombination and repair, cell wall/membrane/envelope biogenesis, energy production and conversion, amino acid transport and metabolism, inorganic ion transport and metabolism, and carbohydrate transport and metabolism. In terms of relative abundance, the three functions of translation, ribosome structure and biogenesis, cell wall/membrane/envelope biogenesis, and energy production and conversion were significantly higher in F0 compared to other samples. After fermentation, there was a significant increase in the relative protein content associated with transcription, amino acid transport and metabolism, inorganic ion transport and metabolism, as well as carbohydrate transport and metabolic functions.

### 3.7 Correlation analysis of the predominant microbes and chemical compounds

**Figure 6:**
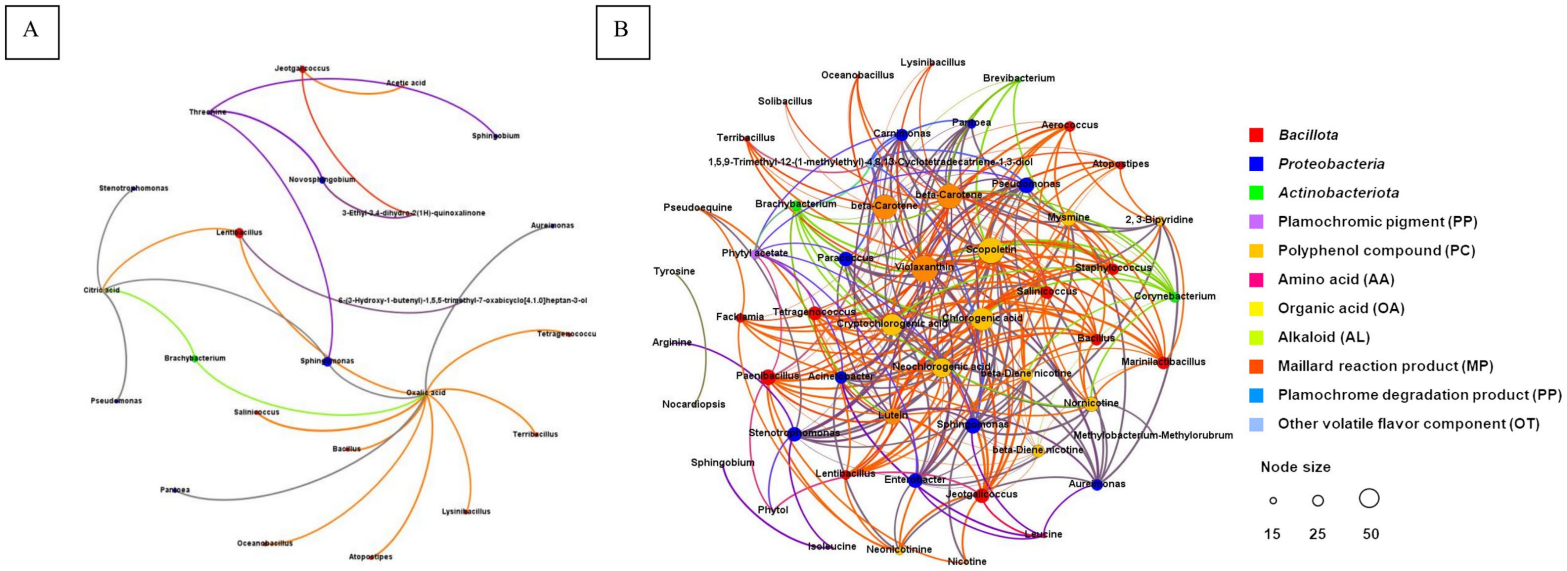
Network of microbes and chemical compositions correlation. The positive **(A)** and negative **(B)** correlations between the microorganisms and chemical composition, respectively.

Spearman’s correlation coefficient (*p* < 0.01) was used to establish the correlation between the top 52 dominant bacteria and 48 chemical components that significantly contributed to the sensory function. There were 48 positive correlations between 17 microorganisms (nine *Bacillota*, seven *Pseudomonadota* and one *Actinobacteriota*) and six chemical components. There were 492 negative correlations between 30 microorganisms (15 *Bacillota*, 11 *Pseudomonadota* and four *Actinobacteriota*) and 21 chemical components, and the number of negative correlations was significantly higher than that of positive correlations (Figure 6). Specifically, oxalic acid significantly and positively correlated with 12 bacterial genera, including *Bacillus*, and *Oceanobacillus*. Citric acid was significantly positively correlated with *Pseudomonas*, *Stenotrophomonas*, *Brachybacterium*, *Sphingomonas*, and *Lentibacillus*, whereas threonine was significantly positively correlated with *Novosphingobium*, *Sphingobium*, and *Sphingomonas*. There were significant positive correlations between 3-ethyl-3,4-dihydro-2(1H)-quinoxalone and *Jeotgalicoccus*, acetic acid and *Jeotgalicoccus*, and (3S,5R,8S,7Z,9ζ)-5, 6-epoxy-7-macrostigmo-3, 9-diol and *Lentibacillus.*

In terms of negative correlation, violaxanthin, scopoltin and *β*-carotene are negatively associated with 26, 25, and 24 bacteria genera respectively, including *Acinetobacter*, *Staphylococcus*, *Corynebacterium*, *Bacillus*, *Oceanobacillus*, and et al. There were 15 strains, such as *Acinetobacter*, *Bacillus*, *Staphylococcus*, and others, exhibited a significant negative correlation with more than 10 components.

## 4 Discussion

This study utilized a gradient temperature change method, in contrast to the conventional industrial fermentation process, to investigate its impact on CTLs quality. Compared to previous studies, we not only measured the conventional components, but also the non-volatile components and absolute quantitative bacterial structure and function in CTLs. The goal was to uncover the underlying mechanism behind the impact of gradient temperature fermentation on CTLs, providing new directions and theoretical support for CTLs fermentation. The results showed that, compared with constant temperature fermentation, the gradient variable temperature fermentation significantly improved the aroma, maturity, softness, cleanliness, and taste sensation of CTLs. The gradient variable temperature fermentation resulted in significant changes in chemical compositions, bacterial community and function of tobacco leaves, but it had no significant effects on the richness and diversity of microbial communities present in the CTLs. Throughout the entire variable temperature fermentation process, the changes of microorganisms and chemical components were multistage, and they did not appear at the same time from the beginning. The gradient variable temperature fermentation was beneficial for the growth of *Corynebacterium*, *Oceanobacillus*, *Bacillus*, and *Staphylococcus*, which played crucial roles in facilitating the growth and maintaining microecology of CTLs. Through carbohydrate metabolism and amino acid metabolism, these four bacterial genera were capable of producing protease and lipase to the production of peptides, amino acids, and fatty acids, catabolizing sugars and polyphenol compounds, and synthesizing amino acid and organic acid. As a result, this process effectively reduced the irritability and impurities present in CTLs.

Compared to unfermented samples, the pH, total sugar, reducing sugar, alkaloid, and chloride ion contents of all fermented CTLs were significantly reduced, which is consistent with the results of previous studies (Jia et al., 2023). After 20th day of gradient temperature fermentation, no total sugar was detected in CTLs, suggesting a more comprehensive transformation of total sugar during gradient temperature fermentation compared to constant temperature fermentation. The reduction of total sugars could be attributed to the growth and metabolism of strains, Maillard reactions between reducing sugars and amino acids, as well as continued enzymatic hydrolysis or hydrolysis of macromolecular substances (Zhu et al., 2023). In the investigation of CTL fermentation, in addition to the relationship between pH changes, conventional chemical components, volatile aroma compounds, and microbial community composition that scholars mainly focused on (Cai et al., 2024; Wang et al., 2024; Zhang et al., 2024), non-volatile constituents, such as plamochromic pigments, polyphenol compounds, and amino acids, have been reported to impact the colour and aroma of CTLs (Zhao et al., 2007; Kou et al., 2013; Lu et al., 2019); the enzymatic Browning reaction between tyrosine and polyphenols (Yang, 2019) was one reason for the decrease in polyphenols and tyrosine in tobacco leaves; amino acids are important precursors of tobacco aroma, and appropriately increasing the amino acid content within a certain range was helpful in increasing the aroma (Liu et al., 2022); organic acids are important constituents of tobacco because they directly influence pH during fermentation (Di Giacomo et al., 2007), thereby affecting microbial growth and the production of related substances, moreover, during cigar burning, organic acids in CTLs could combine with alkaloids to regulate the status and proportion of nicotine in the smoke, thereby affecting the taste and aroma of CTLs (Du et al., 2017). Compared with F0, the plamochromic pigment and polyphenol compound content in F30, T1 and T3 were significantly reduced (Figure 2A), indicating that variable temperature fermentation and low/high temperature constant temperature fermentation were beneficial for the degradation of colour changing pigments and polyphenols, and had a greater impact on CTL colour. It was noteworthy that throughout the process of variable temperature fermentation, the contents of plamochromic pigment and polyphenol showed a significant decrease in F20.

Fermentation could change the content of substances in CTLs, thereby affecting the sensory evaluation of CTLs, and some changes in substances were related to changes in the surface bacteria of CTLs. Changes in the microbial community structure, succession, and function in CTLs were further analysed using a high throughput absolute quantitative method. Compared to conventional relative abundance investigations, this method unveiled more accurate fluctuations in microbial population and function (Tkacz et al., 2018; Tourlousse et al., 2017). Different fermentation temperatures and times together caused significant differences in the bacterial community, but had no significant effects on the richness and diversity of the bacterial community. Co-occurrence analysis showed 1,068 positive and 68 negative correlations in CTLs, which also indicated that variable temperature fermentation had no significant effect on the microecological stability of CTLs (Ma et al., 2020). The COG and KEGG databases were used to compare the functional genes of the bacteria, and both showed that carbohydrate and amino acid metabolism were the main metabolic functions of the CTL samples. During fermentation, the absolute abundance of each bacterial function gradually decreased. However, in terms of relative abundance, amino acid and carbohydrate metabolism increased in the fermented CTLs. During the process of fermentation, *Bacteroidota* decreased the most, and the absolute dominant bacteria in CTLs gradually changed from *Bacteroidota* to *Bacillota* and *Actinobacteria*. At the genus level, the predominant genus of F30, T1, T2, and T3 transitioned from *Acinetobacter* to *Staphylococcus* and *Corynebacterium*, *Staphylococcus* and *Corynebacterium* had protease and lipase activities, which were conducive to the production of peptides, amino acids, and fatty acids (Aro et al., 2010; Tabanelli et al., 2012), and played a role in promoting the formation of cigar tobacco flavour. After 10 days of low temperature fermentation, the microbial abundance decreased from 10^8^ copies to 10^7^ copies, and following 10 days of moderate temperature fermentation, the microbial count further reduced to 10^6^ copies, and remained stable at 10^6^ copies until the end of fermentation (Figure 4A). This once again proved that the number of microorganisms did not constantly change during the variable fermentation process, and it tended to stabilize in the later stages of fermentation. After 30 days of moderate temperature fermentation, it was noteworthy that the bacterial count was 10^7^ copies, which was the highest among all samples at the end of the fermentation process. The reason for this phenomenon might be that moderate temperature fermentation was conducive to the growth of *Staphylococcus* and *Corynebacterium*, while low temperature had an adverse effect on the growth 30–37°C. However, our study suggested that these two bacteria might have a moderate temperature (45°C) as their optimal survival temperature in the complex environment of CTLs, indicating the need for further investigation into the optimal growth and metabolism temperatures of bacteria within this intricate system. Furthermore, the screening and application of these two strains on CTLs should also take into account the use of moderate temperatures.

**Figure 7:**
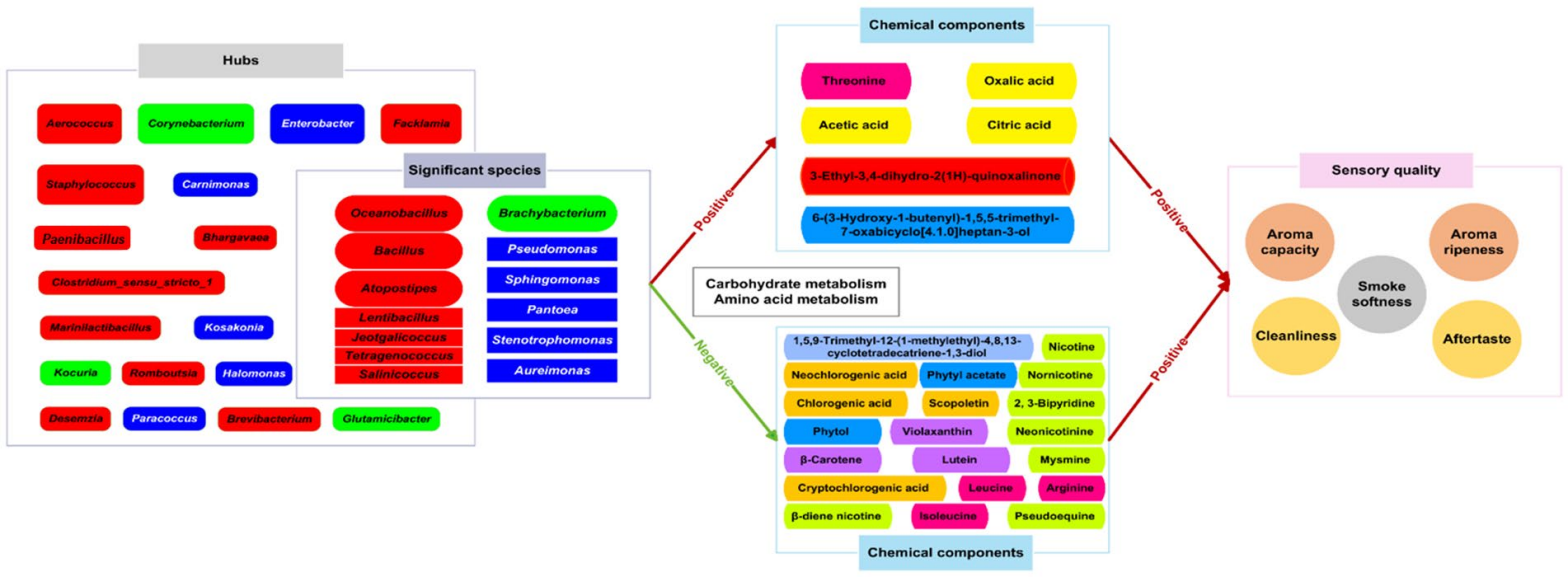
The impact of gradient variable temperature fermentation on the quality of CTLs.

In the process of variable temperature fermentation, the microbial growth and metabolism of CTLs affected the content of chemical substances that were beneficial to the sensory quality of cigar leaves. Correlation analysis showed that 48 chemical components were significantly positively correlated with the sensory quality of CTLs. Among these 48 chemical compositions, 26 were found to have a significant association with bacterial genera. Among the 31 hubs, 13 genera had found to be associated with these 26 substances. Therefore, these 13 bacterial genera, *Atopostipes*, *Aureimonas*, *Bacillus*, *Brachybacterium*, *Jeotgalicoccus*, *Lentibacillus, Oceanobacillus*, *Pantoea*, *Pseudomonas*, *Salinicoccus*, *Stenotrophomonas*, *Sphingomonas*, and *Tetragenococcus*, were defined as important bacterial genera. These bacteria played crucial roles in facilitating the growth and maintaining microecology of CTLs, and synthesizing aromatic compounds. Specifically, *Oceanobacillus* was common endophytic bacteria that promote plant growth (Alhindi and Albdaiwi, 2022). *Brachybacterium* played an important role in maintaining tobacco microecology (Zhang et al., 2020a). *Pantoea* and *Stenotrophomonas* could inhibit plant pathogenic fungi (Yang et al., 2021) and *Acinetobacter*, *Sphingomonas* and *Pseudomonas* screened from tobacco cultivation soils could degrade nicotine (Wang et al., 2011). *Sphingomonas* in tobacco leaves degraded lignin into aroma substances (Masai et al., 1999). Six chemical components, including threonine with a sweet taste, oxalic acid, acetic acid and citric acid that can soften the smoke (Xie, 2009), 3-ethyl-3,4-dihydro-2(1H)-quinoxalinone and 6-(3-hydroxy-1-butenyl)-1,5,5-trimethyl-7-oxabicyclo[4.1.0]heptan-3-ol, were significantly positively correlated with 13 important bacterial genera. And 13 important bacterial genera participated in the changes of chemical substances in CTLs mainly through amino acid metabolism and carbohydrate metabolism (Figure 7). The alterations in these beneficial bacterial genera during the fermentation process had a direct impact on the material composition of CTLs, consequently influencing their aroma and flavor. This study highlighted that gradient variable temperature fermentation was particularly effective in eliciting this advantageous effect. In comparison to constant temperature fermentation at varying degrees, it proved more conducive to regulating the growth and metabolism of bacteria on the surface of Indonesian CTLs, thereby facilitating changes in chemical compounds that contributed to the sensory quality of CTLs.

Overall, a three-phase model could be used to characterize the alterations of CTLs during gradient variable temperature fermentation. Compared to F0, the microbial composition of F10 had undergone significant changes in relative abundance composition at the genus level, with a decrease in the proportion of *Acinetobacter* and an increase in the proportion of *Corynebacterium*, *Stapyhlococcus*, and *Bacillus*. Compared to F10, the proportion of *Corynebacterium* and *Oceanobacillus* increased in F20, while the proportion of *Stapyhlococcus* decreased. The difference in microbial composition (relative abundance) between F20 and F30 was similar. It could be inferred that the F10 stage was mainly the growth stage of microorganisms, while F20 and F30 were mainly the stages of material accumulation and change. Therefore, Phase I (35°C) was the microbial stage, during which there was a significant decrease in both the total number and function of microorganisms. The phylum of bacteria with relative advantage shifted from *Pseudomonas* to *Bacteroidetes* and *Actinobacteria*, and the dominant genera shifted from *Acinetobacter* to *Staphylococcus* and *Corynebacterium*. F10 exhibited chemical components richness, with the contents of five decreased and 10 increased, especially in polyphenol compound. Elevated concentrations of polyphenol compounds led to heightened irritation and diminished sensory quality. Although the decline of microbial populations persisted, a total of 23 chemical components showed significant changes in F20. Therefore, phase II (45°C) marked a chemical stage. There were 17 chemicals significantly decreased and six increased. Through carbohydrate metabolism and amino acid metabolism, *Corynebacterium*, *Oceanobacillus*, *Bacillus*, and *Staphylococcus*, produced protease and lipase to the production of peptides, amino acids, and fatty acids, catabolizing sugars and polyphenol compounds with irritant taste, and synthesizing amino acid and organic acid contributed to the mellowing of smoke, resulting in an enhancement of sensory scores. Phase III (55°C) indicated a relative mature stage with the highest score of sensory evaluation. Eight compositions from plamochromic pigments and polyphenol compounds exhibited gradual decreases. The number of microorganisms remained at 10^6^ copies, with an increase in the proportion of *Bacillus* and *Oceanobacillus*, a gradual decrease in plamochromic pigments and polyphenol compounds, and an increase in the relative content of carbohydrate metabolism and amino acid metabolism. This indicated that the strengthening of microbial metabolic activity at this stage led to a decrease in polyphenol content, resulting a decrease in irritability and smoke softness. On the other hand, it increased amino acid content to enhance tobacco aroma. Therefore, the sensory score of CTL reaches the highest level at F30.

## 5 Conclusion

Gradient variable temperature fermentation improved Indonesian CTLs’ sensory quality and altered bacteria quantity and function without affecting community richness and diversity, compared to constant temperature fermentation. It also reduced sugar and cembrane products while increased amino acids. Changes in the number and function of microbial genera caused changes in the chemical composition of CTLs, ultimately improving their sensory quality of CTLs. The process of gradient variable temperature fermentation underwent three phases: (I) the microbial stage at 35°C for the first 10 days, with a decrease in microorganisms and a shift in dominant genera, from *Acinetobacter* to *Staphylococcus* and *Corynebacterium*, and with high reducing sugar, polyphenol compound and low sensory score. (II) The chemical stage at 45°C for the second 10 days, improved the sensory evaluation by decreasing 17 chemicals and increasing six, while also reducing microbial populations. The enhanced relative abundances of four microecological hubs, *Staphylococcus*, *Corynebacterium*, *Oceanobacillus*, and *Bacillus*, had the potential to produce protease and lipase to the production of peptides, amino acids, and organic acids, and catabolizing sugars and polyphenol compounds, through carbohydrate metabolism and amino acid metabolism. (III) The mature stage at 55°C for the final 10 days, with the highest sensory score. Eight compositions from plamochromic pigments and polyphenol compounds exhibited gradual decreases, while relative contents of carbohydrate metabolism and amino acid metabolism increased. The results of this investigation provided valuable technical and theoretical support for improving the cigar fermentation process and enhancing the overall quality of CTL.

## Data Availability

The original contributions presented in the study are included in the article, further inquiries can be directed to the corresponding author. Metagenomic sequencing data in this study are deposited in the NCBI BioProject repository accession number PRJNA1163032.
