# Supplementary material for: The impact of gradient variable temperature fermentation on the quality of cigar tobacco leaves
Source: Front Microbiol. 2024 Dec 13;15:1433656. doi: 10.3389/fmicb.2024.1433656 (PMC11672604; doi:10.3389/fmicb.2024.1433656)
Supplement: Supplementary file 1 [file Data_Sheet_1.pdf]

Supplementary Table 1 The chemical composition of CTLs†

Compounds	No.	samples						
		F0	F10	F20	F30	T1	T2	T3
Neoxanthin	PL-1	2.15±0.14a	2.10±0.03a	0.07±0.03c	0.04±0.01c	2.01±0.10a	2.36±0.35a	1.29±0.88b
Violaxanthin	PL-2	0.90±0.29a	0.79±0.09ab	0.62±0.05bc	0.36±0.10d	0.73±0.08abc	0.81±0.06ab	0.50±0.13d
Lutein	PL-3	177.89±13.47a	179.76±2.25a	132.75±0.97c	111.77±1.76de	130.48±2.16cd	157.66±13.96b	97.62±20.76e
Chlorophyll B	PL-4	0.97±0.06a	0.69±0.03b	0.17±0.00d	0.18±0.02d	0.18±0.01d	0.36±0.09c	0.21±0.04d
Chlorophyll A	PL-5	2.38±0.73a	1.59±0.40abc	1.48±0.40bc	0.79±0.45c	1.32±0.53bc	2.17±0.25ab	1.37±0.18bc
β-Carotene	PL-6	19.48±2.19b	21.61±0.42ab	20.50±0.59b	15.79±0.49c	21.00±1.05ab	24.30±3.33a	16.17±2.46c
Neochlorogenic acid	PO-1	132.88±5.74b	155.47±11.25a	116.97±2.10c	95.99±2.42d	119.05±6.46c	114.60±11.73c	94.65±5.52d
Chlorogenic acid	PO-2	961.69±8.26b	1151.77±4.26a	603.78±6.39e	528.57±24.26f	645.13±25.96d	884.54±35.01c	479.83±34.11g
Cryptochlorogenic acid	PO-3	299.35±10.34b	326.58±16.38a	254.09±6.88c	220.22±13.01d	255.37±10.66c	272.79±12.03c	217.14±9.73d
Scopoletin	PO-4	82.81±2.28bc	99.28±2.82a	73.46±0.34d	64.91±1.00f	79.73±0.25c	86.04±2.15b	69.20±4.18e
Rutin	PO-5	1156.76±11.49b	1248.96±16.19a	236.88±2.52d	199.00±8.83e	252.41±2.09d	949.50±16.73c	152.12±9.18e
Aspartic acid	AA-1	11706.95±1945.17a	10639.33±2237.74ab	11519.26±937.64a	11393.23±920.00a	7535.72±3353.05bc	8849.82±464.51abc	7255.91±665.43c
Serine	AA-2	2669.67±230.93a	3000.37±81.37a	2845.41±107.27a	2518.24±131.62a	1846.22±575.97b	2643.82±379.21a	1987.83±102.87b
Glutamic acid	AA-3	1297.91±439.81	1034.37±204.58	802.34±220.94	1042.33±153.41	691.48±201.66	1207.27±715.06	769.66±73.57
Glycine	AA-4	94.42±38.97c	252.33±37.51b	402.02±81.19a	368.17±57.66a	95.26±46.46c	85.92±16.64c	84.77±41.07c
Histidine	AA-5	426.84±22.50c	575.56±8.29bc	621.85±118.08b	591.30±121.46bc	653.98±69.36b	830.47±169.82a	661.85±35.24b
Arginine	AA-6	240.02±22.54a	245.21±11.06a	224.14±10.86ab	213.92±12.66ab	148.35±99.73b	235.86±36.13a	180.23±14.62b
Threonine	AA-7	42.89±8.08ab	30.28±16.62b	62.11±2.82a	43.00±17.23ab	62.15±18.63a	39.76±9.45ab	57.11±6.11a
Alanine	AA-8	154.36±9.12	159.30±24.16	186.06±15.68	157.68±29.48	144.42±33.73	185.92±39.13	140.46±36.40
Proline	AA-9	893.77±134.44ab	686.12±85.95bc	838.13±41.88abc	599.52±19.04c	722.77±125.19abc	974.26±256.02a	779.73±206.18abc
4-Aminobutyric acid	AA-10	4456.75±680.58b	6174.89±215.28a	4870.25±157.47ab	4565.16±381.16b	3782.99±1607.37b	4528.53±903.71b	3364.60±381.29b
Cystine	AA-11	28.34±21.82	39.51±1.84	26.93±14.70	32.20±2.47	18.88±19.52	40.64±5.51	16.29±9.33

Tyrosine	AA-12	63.96±53.11b	117.83±7.37a	128.36±11.36a	108.73±10.09a	151.02±3.74a	139.07±14.26a	128.43±5.01a
Valine	AA-13	72.00±21.91ab	88.45±1.15a	81.19±8.49a	83.32±11.96a	50.20±19.29bc	81.18±12.58a	47.26±4.49c
Methionine	AA-14	36443.93±3461.95	42209.82±2427.46	59630.75±4485.08	58929.22±4511.82	41484.65±35152.66	23056.11±30229.98	45491.24±37610.34
Lysine	AA-15	86.12±15.66	84.66±2.34	91.35±15.14	92.92±18.56	80.39±22.92	70.97±12.43	84.03±6.82
Isoleucine	AA-16	104.61±11.73b	128.18±2.70a	92.27±3.42b	88.45±10.44b	62.92±17.26c	95.31±9.20b	59.27±3.18c
Leucine	AA-17	279.44±32.07a	280.71±3.46a	172.18±7.09c	162.18±3.84c	135.32±33.10cd	218.42±26.79b	121.95±1.10d
Phenylalanine	AA-18	5481.07±3089.10ab	3394.50±237.67b	5045.41±1297.62ab	3041.95±2634.58b	10192.55±3588.27a	6583.49±932.56ab	6524.67±5718.22ab
Oxalic acid	OA-1	32.64±0.25a	33.28±1.93a	34.70±1.50a	35.76±0.69a	27.91±5.95b	33.97±2.02a	35.88±1.04a
Tartaric acid	OA-2	0.02±0.00	0.02±0.01	0.02±0.01	0.02±0.01	0.02±0.01	0.02±0.01	0.02±0.01
Formic acid	OA-3	5.16±0.29c	6.61±1.11c	17.53±1.82b	18.80±0.74b	17.85±2.93b	6.09±0.48c	21.51±1.26a
Malic acid	OA-4	23.34±0.89ab	22.79±2.57ab	22.71±2.92ab	24.97±1.00ab	22.24±2.11b	25.16±1.11ab	26.05±1.81a
Malonic acid	OA-5	1.04±0.27	1.63±1.26	2.31±1.90	0.94±1.32	1.93±1.74	2.05±0.46	1.24±1.72
Ketovaleric acid a	OA-6	0.19±0.02	0.19±0.04	1.45±2.03	3.28±2.59	2.01±3.07	0.29±0.03	3.89±3.06
Lactic acid	OA-7	3.62±0.58bc	3.27±0.11c	4.66±1.27bc	4.45±0.37bc	3.40±0.27bc	7.02±0.60a	4.91±1.39b
Acetic acid	OA-8	23.85±1.66d	24.80±6.20d	48.76±6.81bc	55.93±2.40bc	46.91±4.93c	83.36±4.18a	55.15±3.11b
Citric acid	OA-9	8.46±1.02c	10.35±2.16abc	11.09±1.82abc	11.81±1.58ab	10.21±1.67abc	9.15±0.49bc	12.56±1.40a
Maleic acid	OA-10	1.00±0.06	0.93±0.20	0.97±0.08	0.99±0.02	0.90±0.00	1.02±0.04	0.89±0.09
Fumaric acid	OA-11	10.49±0.73bc	9.73±1.65c	11.36±0.94abc	12.09±0.34ab	10.37±1.97bc	12.73±0.16a	11.76±0.30abc
Succinic acid	OA-12	7.06±2.18	6.38±1.00	8.16±0.74	7.96±0.31	6.33±1.17	7.93±0.12	6.82±0.53
Nicotine	AL-1	16.30±2.24a	14.33±0.19ab	12.66±0.29b	13.11±0.19b	13.39±0.39b	11.73±3.23b	12.80±0.29b
Nornicotine	AL-2	3889.15±440.31a	3877.38±194.34a	2984.44±92.80bc	2956.71±162.70bc	3048.07±106.45bc	3282.10±266.55b	2753.42±138.33c
Myosmine	AL-3	99.27±13.08a	101.74±7.05a	82.24±2.86cd	74.12±2.41de	85.75±0.99bc	96.57±3.72ab	69.34±4.65e
Pseudoequine	AL-4	193.96±20.51a	178.31±1.73a	146.98±43.53ab	96.49±1.31b	151.09±46.69ab	149.57±43.37ab	174.50±2.60a
β-diene nicotine	AL-5	15.49±1.67a	11.37±1.83b	6.62±0.60c	6.36±0.28c	8.48±0.19c	11.81±1.29b	8.19±0.98c
Anatabine	AL-6	1213.11±156.37a	1131.18±10.71ab	1045.54±18.47b	1021.49±15.28b	1052.61±28.54b	1061.05±39.81b	1002.57±56.91b
2, 3-Bipyridine	AL-7	95.31±11.02a	82.57±0.81b	66.65±0.78cd	64.07±1.03d	73.11±1.06c	81.50±1.40b	60.32±3.38c

Cotinine	AL-8	81.77±26.38	80.44±2.85	82.01±0.73	78.06±0.84	78.11±10.99	72.02±9.18	71.08±10.27
4-Cyclopentene-1,3-dione	MP-1	-	-	16.53±0.81	17.78±1.64	16.17±2.05	-	14.58±0.77
3-Methylpentanoic acid	MP-2	89.13±4.56a	76.73±5.83b	72.15±3.25bc	68.17±6.63bc	69.67±7.09bc	54.84±10.97d	60.22±3.06cd
3-Ethyl-3,4-dihydro-2(1H)-quinoxalinone	MP-3	120.62±9.84d	148.26±17.03c	177.01±5.75ab	177.83±11.10ab	196.14±23.24a	161.91±3.45bc	176.17±6.86ab
Solanone	CP-1	29.38±2.10a	25.54±2.59ab	21.97±1.97bc	19.59±1.81c	19.49±3.18c	18.45±2.17c	14.30±2.13d
Thunbergol	CP-2	132.86±60.15	68.27±28.38	65.25±60.69	66.13±59.78	98.20±65.75	91.75±71.69	105.40±50.05
Dihydroactindiolide	PP-1	28.18±1.83a	8.35±14.46b	-	-	-	30.32±3.79a	-
Megastigmatrienone	PP-2	50.31±3.61a	53.96±6.54a	23.33±10.20b	26.83±11.33b	20.36±4.50b	48.59±0.71a	7.79±0.67c
4-(3-Hydroxy-1-butenyl)-3,5,5-trimethyl-2-cyclohexen-1-one	PP-3	19.22±1.74a	17.74±1.26a	-	3.54±6.13b	-	4.08±7.07b	-
6-(3-Hydroxy-1-butenyl)-1,5,5-trimethyl-7-oxabicyclo[4.1.0]heptan-3-ol	PP-4	81.75±2.44bc	80.90±6.62c	97.01±4.28a	97.05±4.35a	95.91±13.15ab	93.13±10.03abc	88.63±7.75abc
6-Hydroxy-4,4,7a-trimethyl-5,6,7,7a-tetrahydrobenzofuran-2(4H)-one	PP-5	51.25±4.03	47.02±6.25	45.27±6.88	46.96±1.23	51.64±5.51	47.45±2.26	46.53±2.26
6,10,14-Trimethyl-2-pentadecanone	PP-6	33.57±2.44ab	36.49±5.54a	33.10±3.15ab	31.39±5.15ab	35.02±4.42a	36.13±1.93a	27.51±1.72b
phytyl acetate	PP-7	677.50±43.62ab	744.17±68.91a	622.92±2.08bc	609.08±28.90bc	639.92±66.91bc	734.33±31.23a	568.50±18.50c
Phytol	PP-8	308.83±18.11ab	333.92±25.84a	300.17±1.53b	256.09±13.89cd	280.67±26.76bc	283.25±14.28bc	240.90±9.33d
2,4-Dimethyl-1-heptene	OT-1	47.97±11.22	44.66±16.82	42.19±6.87	46.73±8.81	46.21±27.27	54.15±5.45	44.27±23.32
2-Methylbutanoic acid	OT-2	-	-	2.61±4.52b	-	10.24±1.85a	2.46±4.26b	-
3-Methoxy-3-methyl-1-butanol	OT-3	2.35±4.06	-	-	5.22±4.73	6.27±5.75	3.34±4.34	5.95±5.18
Hexadecanoic acid, methyl ester	OT-4	4.06±7.04	6.94±12.02	-	-	-	12.14±1.55	-
1,5,9-Trimethyl-12-(1-methylethyl)-4,8,13-cyclotetradecatriene-1,3-diol	OT-5	1962.63±208.53ab	2201.31±253.75a	1864.22±29.23b	1801.89±114.44b	1964.73±190.51ab	2046.99±33.21ab	1482.89±79.29c

Isoaromadendrene epoxide	OT-6	56.38±48.93	56.19±48.66	47.47±41.70	-	94.58±90.05	67.37±58.38	82.13±20.88
Sclareolide	OT-7	-	-	-	-	27.29±47.27b	61.35±1.86a	-

Note: † The chemical compounds contents were measured in μg/g, with the exception of organic acids and nicotine units which were measured in mg/g. "-" meant not detected. Different letters indicated significant differences between different groups ($P < 0.05$). F0, F10, F20, and F30 were subjected to fermentation at gradient variable temperatures over the course of 0, 10, 20, and 30 days. T1, T2, and T3 underwent fermentation at consistent low, moderate, and high temperatures for a duration of 30 days.
